# The Effect of Offline Medical Resource Distribution on Online Physician-Patient Interaction: Empirical Study With Online and Offline Data

**DOI:** 10.2196/43533

**Published:** 2023-01-10

**Authors:** Shanshan Guo, Yuanyuan Dang, Doug Vogel, Bofei She

**Affiliations:** 1 School of Business and Management, Shanghai International Studies University Shanghai China; 2 School of Business Administration, South China University of Technology Guangzhou China; 3 School of Management, Harbin Institute of Technology Harbin China

**Keywords:** medical resources, online health community, physician-patient interaction, online and offline, social network analysis

## Abstract

**Background:**

The relationship between online health communities (OHCs) and offline medical care is unclear because both provide physician-patient interaction services and channels. Taking advantage of information and communication technology, patients have been using OHCs widely. However, some physical medical resources (such as hospital beds and medical devices) cannot be replicated by information and communication technologies. Therefore, it is worth studying how offline medical resources affect physician-patient interactions in OHCs and how OHCs help to solve resource scarcity and the uneven distribution of traditional medical treatment.

**Objective:**

This study aimed to support the notion that physician-patient consultations in OHCs are influenced by the objective distribution of offline health care capital (accessibility and availability) and to provide suggestions for the allocation of medical resources in practice through the judicious use of offline and online channels.

**Methods:**

The empirical data in this study were collected from both online and offline channels. The offline data include 9 years (2006-2014) of medical resource statistics of 31 provincial administrative regions in mainland China. Moreover, data regarding the geolocation-based physician-patient interaction network in the OHC were also collected. The online data come from one of China’s largest OHCs. We obtained 92,492 telephone consultation records of 6006 physicians using an automatic web crawler program. Social network analysis was used to visualize the descriptive statistics of the offline geolocation-based physician-patient interaction network in the OHC. A regression model with a squared variable was applied to analyze online and offline empirical data to further test our hypothesis. Two types of robustness tests were used to increase the reliability of the test results of the initial model.

**Results:**

The results of our social network analysis show that there is a uniform geographic distribution of patients who use OHCs, whereas the physician relies more on geographic advantage (eg, a higher medical resource capability). Moreover, the empirical results of the regression model support the notion that physician-patient telephone consultations are positively influenced by physicians’ online contributions (β_contribution_=.210; *P*<.001) and capital availability (β_bed_=.935; *P*=.07), and, interestingly, spatial accessibility has an inverted U–shaped effect (β_distance_=.199; *P*<.001 and β_distance_^2^=–.00449; *P*=.008). The results indicate that the use of OHCs, although constrained by offline medical resources, provides a channel for offline resources to flow from areas with high availability to those with low availability.

**Conclusions:**

This study explores the relationship between online and offline channels by investigating online physician-patient interactions and offline medical resources. In particular, this study analyzes the impact of offline channels on online channels and verifies the possibility of OHC capital use shifting from a high-availability area to a low-availability area. In addition, it provides a theoretical and practical basis for understanding the interaction of online and offline channels of medical care.

## Introduction

### Background

Health care is one of the most critical elements of human life, and as the population ages, and the prevalence of chronic diseases increases, people’s health awareness increases. Balancing the increasing demand and scarce medical resources in health care has become a global challenge. Online health communities (OHCs), which use information and communication technology, are believed to be an essential tool for addressing this challenge and key to achieving pleasing, ongoing physician-patient interactions [1]. With advances in information and communication technology, the use of the internet has decreased the cost of communication [2]. Some even point out that the internet has eliminated distance limitations [3] and created a “flat world” [4].

OHCs embody the nonregional nature of a network, enabling the network to break the geographic bottleneck of traditional health care. In addition, several scholars have demonstrated that OHCs play significant roles in information dissemination, emotional support, knowledge sharing, and so on [5-10]. Nevertheless, although the relationship between OHCs and offline medical resources (eg, hospitals, testing equipment, and physicians) has been growing slowly, it is easily depleted, difficult to replicate, and highly exclusive. Therefore, it is seldom considered. In fact, research on online and offline channels shows that they are complementary and synergistic [11].

Ways to rationally allocate and improve the efficiency of offline medical delivery with limited resources to provide maximum health care and health management support to individuals are a persistent topic of traditional health care statistical research [12]. Related research has focused on improving health care resource distribution in terms of spatial accessibility and capital availability [13-15]. Spatial accessibility refers to the geographic availability and accessibility of health care resources and is generally measured by using distance methods (eg, linear spatial distance, network distance, and average elapsed time) [13]. As part of spatial equity studies, capital availability refers to the average distribution of resources. It describes the amount of health care capital available per capita, as measured by the number of objective forms of health care resources (beds, physicians, and nurses) per 1000 population. In traditional studies, the rational allocation of offline medical resources, such as the spatial (geographic) layout [13] of medical resources, is often considered, whereas the complementary role of online channels is usually ignored. Especially for accessibility (geographic distance), the allocation of offline resources shows a considerable impact compared with online interactions [16].

In recent years, the emergence of COVID-19 has provided a large growth opportunity for OHCs. Several recent studies have shown that for online medical services (including consultations), trust in the health care provider positively affects the use and effectiveness of OHCs [17-19]. There is evidence that the COVID-19 pandemic has led to an improvement in the physician-patient relationship, including an increase in trust [19], thereby providing an impetus to use OHCs. Before the COVID-19 pandemic, eHealth use was low, and only approximately 10% of people communicated online with physicians or had online medical appointments [20]. However, during the COVID-19 pandemic, the spatial accessibility of limited medical resources became a challenge [21,22]. In several countries, such as the United Kingdom and Uganda, there has been an increase in the use of online health services, which are seen as a good method to mitigate reduced access to offline health care resources [21]. Although recent evidence suggests that the geographic distribution of quality hospitals remains unequal in China, OHCs have mitigated the inequity of medical resources between rural and urban areas [22]. The use of OHCs has been influenced by the spatial distribution and accessibility of medical resources, and the COVID-19 pandemic has provided an ideal opportunity to develop OHCs. However, previous studies have not considered the specific impact of the distribution of offline medical resources on the use of OHCs. Therefore, it is necessary to explore the impact of the distribution of offline medical resources on the social exchange between physicians and patients in OHCs. This knowledge will help to leverage the interaction between online and offline channels to optimize the allocation of medical resources so that medical resources are used most appropriately by health care stakeholders.

Hence, this study uses online (OHC) and offline (offline medical resources) data to explore the impact of medical resource distribution on online physician-patient interactions. This research supports the notion that physician-patient consultations in OHCs are influenced by the objective distribution of offline health care resources, and this issue is studied in terms of both spatial accessibility and capital availability. The results of our study provide suggestions for how to allocate medical resources in practice through the judicious use of offline and online channels.

### The Impact of the Spatial Accessibility of Offline Medical Resources on Online Physician-Patient Interactions

It is difficult and expensive to move offline medical resources such as hospital buildings, equipment, beds, and long-tenured physicians, as is the case with most tangible resources. Therefore, the geographic distance between offline medical resources and patients will directly influence the accessibility and availability of these resources. Furthermore, the geographic distribution of spatial accessibility not only affects the use of resources in offline channels but also affects online channels; for example, some empirical studies have pointed out that online channels become more valuable when consumers are farther away from offline markets [16]. Hence, online channels will play a more prominent role, specifically in terms of consumer engagement, as offline spatial accessibility decreases (as distance increases) [2]; for example, a patient in Shenyang, the capital of China’s Liaoning province, who wants to communicate with a physician working at a tertiary hospital in Beijing, almost 700 km away, may choose to have a telephone consultation provided by the OHC because of geographic distance, eliminating travel costs and saving commute time.

Nevertheless, prior studies indicate that although online channels can compensate for the lack of offline spatial accessibility, local familiarity [23,24] cannot be ignored. Molitor et al [25] studied residents purchasing health care services (eg, cosmetic dentistry and osteopathy) via the internet and showed that long distances reduce consumers’ purchase intentions. Meanwhile, other studies have suggested that users’ online search behavior follows the local familiarity effect, that is, users will limit themselves to a geographic search range, and the results beyond this range will not be considered [26]. The local familiarity effect also applies to access to health care resources. As health care is time sensitive, patients are more likely to interact online with nearby physicians in anticipation of a future face-to-face encounter. Furthermore, patients likely have better insight into the medical resources of their surrounding area, such as which hospital or physician is better, including for a particular specialty. Given the local familiarity effect, patients are more likely to have an online physician-patient interaction with nearby physicians to avoid social exchange involving low offline accessibility.

In summary, this study suggests that the probability of having social exchange with physicians will increase when accessibility increases within a certain area. For the area beyond, the probability will decrease when accessibility decreases. On the basis of the aforementioned discussion, we propose hypothesis 1: the frequency of online physician-patient interactions (via telephone consultations) is influenced by the spatial accessibility of offline medical resources, and the effect has an inverted U shape.

### The Effect of the Capital Availability of Offline Medical Resources on Online Physician-Patient Interactions

In addition to spatial accessibility, the availability of offline medical resources is another index of medical resource distribution. In terms of macro resource allocation, spatial accessibility refers to the distance to the nearest available resource. Availability encompasses the ability of high-quality resources to serve users in a geographic area, that is, per capita occupancy. As mentioned earlier, medical resources are characterized by their exclusivity. Different customers cannot use a single medical resource. Therefore, as measured by per capita occupancy, the exclusivity of medical resources is considered when determining the availability of capital; for example, suppose that there are 3 tertiary hospitals within an area in region A, but there is only 1 tertiary hospital in the same size area in region B. Compared with the residents in region B, those in region A have a shorter average distance to reach a tertiary care hospital; therefore, they have higher spatial accessibility. However, region A, which has 3 million people, is more densely populated than region B, which has only 0.5 million people. As a result, the capital availability of region A is 1 hospital per million population, whereas for region B, it is 2 hospitals per million population. Thus, the availability of tertiary hospitals in region B is better than that in region A. For a patient, a lack of spatial accessibility means higher costs (eg, time and money) to reach the nearest hospital (or other health care resource) [16]. Insufficient capital availability implies that a patient will have to share health care resources (eg, hospital beds and physicians) with more patients, which may result in longer wait times during the visit or increased costs because of insufficient supply. These scenarios illustrate that spatial accessibility and capital availability are 2 complementary measures for the rational distribution of medical resources and that the absence of either decreases the likelihood of an effective and reasonable allocation of medical resources.

The inequality in access to health care resources among people in different regions was described by Goh et al [27] as “the health disadvantage of location.” The most typical example of this concept is the inherent difference in the availability of health care resources between urban and rural areas. The main sources of this variation are the social, economic, and environmental differences between these settings [28]. This issue is difficult to solve quickly. In China, tier 1 urban areas (eg, Beijing, Shanghai, and Guangzhou) have the highest medical resource availability [29]. Consequently, there have been population movements to urban areas, especially for people with medical needs [30]. People travel considerable distances from their permanent residences to cities with higher capital availability to obtain superior medical resources.

The emergence of OHCs provides more opportunities to support offline interactions between physicians and patients, such as referrals (physicians can recommend offline face-to-face treatment when OHCs cannot meet medical needs) and routine telephone consultations to keep in touch as part of the offline physician-patient relationship. Hence, for patients who seek quality medical resources, especially in regions different from their current areas of residence, the larger the difference in capital availability, the more inclined they will be to engage in online interactions with a physician located in their destination region. Therefore, based on the aforementioned discussion, we propose hypothesis 2: the frequency of online physician-patient interactions (by means of telephone consultations) is positively correlated with the variation in offline medical resource availability.

### The Interaction of Spatial Accessibility and Capital Availability of Offline Medical Resources

As spatial accessibility and capital availability are 2 independent but complementary measures of the distribution of medical resources, this study suggests that a complementary effect on online physician-patient interactions exists between these 2 indicators. Specifically, the accessibility and availability of offline medical resources have a positive moderating effect on the frequency of physician-patient interactions in OHCs.

There is a significant status difference between physicians and patients in physician-patient interactions. Furthermore, this relationship is often life critical and entails responsibility, obligation, and trust [31]. Physician-patient interactions follow social exchange theory and are understood in terms of “rewards and costs” from an economic and utilitarian perspective [32,33]. During the social exchange, people are more likely to engage in social exchange with lower costs and higher rewards [16]. When there is limited accessibility to offline medical resources that are located outside the patient’s *neighborhood*, the physician-patient interaction is less defined [34], and the patient may pay more in terms of time or even financial costs when offline communication is needed.

For this reason, patients are more likely to forgo this online social exchange because of the trade-offs between the gains and losses of the social exchange. However, suppose that higher-quality health care resources are available to patients when the target region of the physician has higher medical resources. In that case, this availability increases the reward of physician-patient social exchange and consequently increases the likelihood that the patient will choose to engage in an online social exchange with that physician. Similarly, when there is poor availability of medical resources in the physician’s target region, patients will choose not to have an online social exchange with this physician because of long wait times and excessive expenses. When the patient is physically closer to the physician, this proximity reduces the patient’s costs caused by geographic distance and compensates for poor capital availability, thus increasing the possibility of a physician-patient online social exchange.

In summary, this study hypothesizes that the accessibility and availability of offline medical resources can compensate for their respective deficits and complement each other to influence online physician-patient interactions. Therefore, we propose hypothesis 3: the frequency of online physician-patient interactions (via telephone consultations) is positively correlated with the interaction between the spatial accessibility and capital availability of offline medical resources.

## Methods

### Introduction of Online and Offline Data

**Figure 1:**
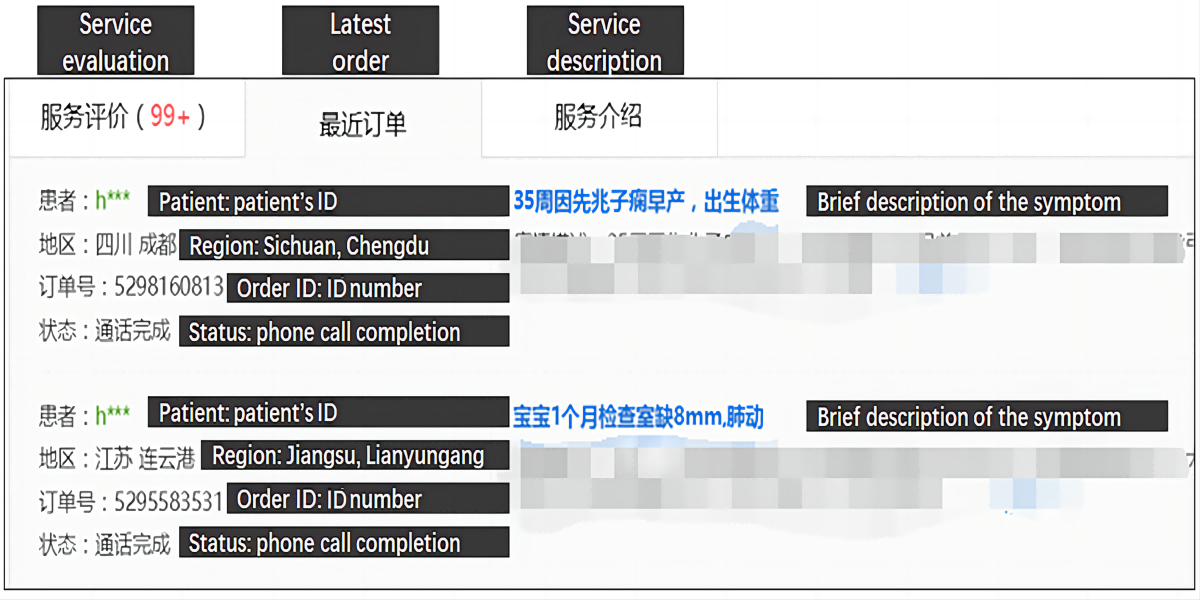
Example of a physician’s recent telephone consultation.

The data in this study are divided into 2 parts: online data and offline data. The offline data, which include data from 2006 to 2014 from the China Health Statistical Yearbook 2014 as well as the latitude and longitude coordinates of the 31 provincial administrative regions in mainland China, describe the distribution of offline medical resources in China. The online data were collected from one of the largest OHCs in China by an automatic web crawler program. These data include the personal information (eg, names, titles, hospitals, departments, and contributions) of all the physicians who provided telephone consultation services on the platform from its establishment until September 2014, as well as the details of all telephone consultations. Importantly, the OHC protects patient privacy by encrypting the patient ID. However, patient locations and a description of disease-specific symptoms are displayed on the site with permission. This information enables physicians to better understand the patient’s history before the telephone consultation to improve the interaction and give patients a sense of authenticity, thereby increasing the number of users. Figure 1 depicts the order information of a recent telephone consultation. The detailed description of the patient’s symptoms has been blurred for confidentiality reasons.

After obtaining the OHC data, we initially cleaned and preprocessed them. Because of both privacy reasons and the display mechanisms of the OHC, only telephone consultations in the last month could be shown. After parsing the information captured by the web crawler, we learned that 6006 physicians had had telephone consultations with patients. This figure accounts for 22.4% (6006/26,813) of all physicians who had provided telephone consultation services. There were 92,492 telephone consultations, with an average of 15.4 (SD 75.86) telephone consultations per physician in the last month.

Next, the offline medical resource data were processed. This study uses offline data sourced from China’s 2014 public health statistics in combination with the online data from the study period of the last month. The offline data included statistics from different provincial regions, such as the number of beds in hospitals and health centers per 1000 population, general hospital beds per 1000 population, and full-time physicians per 1000 population. In addition, an adjacency matrix between provinces was calculated using the central distance.

### Ethical Considerations

As the online data set is composed of crawl data concerning actual physician-patient telephone consultations, and these consultations did not take place for research purposes, the data set is considered a public domain data set and is thus exempt from institutional review board approval as publicly available social media data per Article 27 of the *Data Security Law of the People’s Republic of China*. The automatic crawler program followed the Robots Exclusion Protocol, which is considered a common specification in the internet field. In addition, in accordance with the *Data Security Management Measures* of China, we limited the number of concurrent programs to a single source of web pages to avoid a heavy data burden. We also took precautionary measures in handling the OHC data set because of its sensitive nature [7,8]. On the basis of the privacy policy of our target OHC platform, the patient ID is partially hidden (only the first character is retained, and asterisks replace the rest). The home page example shows only the publicly available information of the physician, and the patient’s user- generated online content (such as the medical process description in the thank-you note) was automatically anonymized. After dealing with the data set, we submitted the research project ethics review application to the institutional review board of the Shanghai International Studies University’s School of Business and Management, which approved the study as *exempt* (review number 2022BC024). Accordingly, the data collection of this study complies with medical ethical norms and requirements.

### Data Description Based on Social Network Analysis

**Figure 2:**
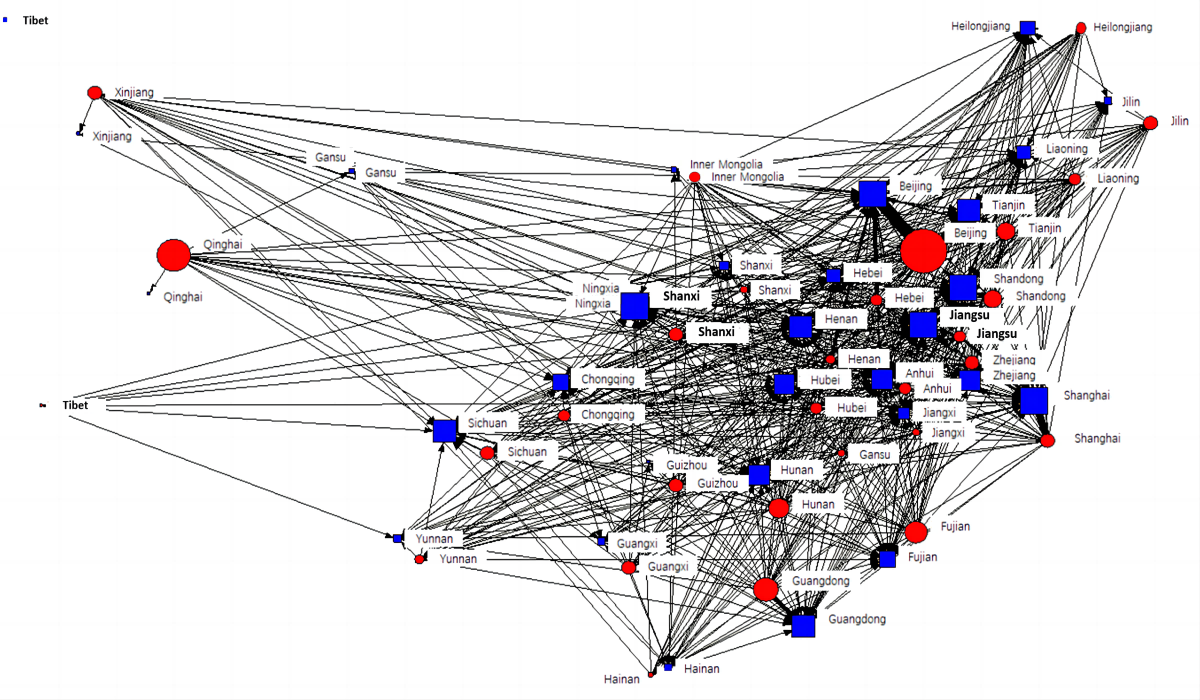
The physician-patient interaction network in the Chinese online health community is based on an offline geographic location in mainland China.

To preliminarily explore whether the physician-patient online interaction would be influenced by the offline channel, we used social network analysis to visualize the descriptive statistics of the offline geolocation-based physician-patient interaction network in the Chinese OHC (Figure 2). In our network analysis, each vertex represents a provincial region. On the basis of the latitude and longitude of their relative locations, these vertices roughly correspond to their actual geographic locations. As the data available from the OHC are limited, the geographic location information of patients is available only for telephone consultations. Other functions and services (such as online consultations or patient communities) do not provide patients’ geographic locations. Therefore, this study uses the frequency of physician-patient telephone consultations to represent the vertices’ online physician-patient interaction attributes. This social network analysis is a directed graph based on offline geographic locations; for example, what is meant when the Beijing vertex points to the Heilongjiang vertex is very different from what is meant when the Beijing vertex points to the Heilongjiang vertex. The latter means that a physician in Beijing has a social exchange with a patient in Heilongjiang, whereas the former means that a patient in Beijing has a consultation with a physician in Heilongjiang. The blue squares represent physicians, specifically the in-degree of the provincial administrative region. The red dots represent patients, specifically the out-degree of the provincial administrative region. The sizes of the blue squares and red dots represent the frequency of interactions: the larger the square, the more telephone consultations a physician in that provincial region has had with a patient.

Figure 2 shows significant differences in the in-degree and out-degree of different provincial administrative regions. In other words, the online physician-patient interactions in each provincial administrative region are not independent of geographic location and follow a completely random distribution. This finding suggests that a new physician-patient interaction network that is completely independent of offline resource distribution cannot be formed.

To further understand the difference in the individual vertices in both the network and overall distribution of online physician-patient interactions, we conducted a statistical analysis based on the relative (in-degree or out-degree) centrality and absolute (in-degree or out-degree) centrality of different provincial regions as well as the (in-degree or out-degree) centralization of the overall network.

Degree centrality is measured by the total number of other actors in the network who are directly linked to an actor of interest. It represents the closeness of connections with others, and a higher value indicates a more central position of the vertex in the network. Degree centrality reflects an actor’s centrality in the network, which implies the ability of its related vertex to influence the other vertices in the network. Formulas (1) and (2) [35] are as follows:

C*_D,c_*(*n_i_*)=*d_c_*(*n_i_*)=Σ*_j_x_ij_* (1)

C*_D,r_*(*n_i_*)=*d_r_*(*n_i_*)=Σ*_j_x_ij_* (2)

where C*_D,c_*(*n_i_*) and C*_D,r_*(*n_i_*) represent the out-degree and in-degree of the nth vertex, respectively. If *x_ij_*=0, it means that there is no direct connection from vertex *n_i_* to vertex *n_j_*, whereas if *x_ij_*=1, it means that a direct connection exists between them. Similarly, if *x_ji_*=0, it means that there is no direct connection from vertex *n_j_* to vertex *n_i_*, whereas if *x_ji_*=1, it means that a direct connection exists between them.

Relative degree centrality is computed in another way using formula (3) [35] to measure the relative centrality of a certain vertex:

*C'_D_*(*n_i_*)=*C_D_*(*n_i_*)/*N*–1 (3)

where *N* represents the total number of the vertices in the network, and *C_D_*(*n_i_*), specifically C*_D,c_*(*n_i_*) or C*_D,r_*(*n_i_*), is the absolute degree centrality of vertex *n_i_*.

Table 1 shows that the top 4 provincial administrative regions ranked by in-degree centrality are Beijing, Shanghai, Jiangsu, and Guangdong. In-degree centrality describes the local central index of these 4 regions and implies that the physicians in these regions enjoy a major advantage during web-based physician-patient social exchange. In addition, in reference to the distribution of offline health care capital, these regions are considered to have high medical care standards in China, as stated in the China Health Statistical Yearbook 2014. Furthermore, these 4 regions hold the top positions in the rankings in terms of the number of tertiary hospitals and general hospitals. This statistical result supports the hypothesis that objective offline medical resources influence the web-based social exchange between physicians and patients. Network centralization measures the degree of concentration of all vertices in the overall level of the network. The network is more concentrated when the value of network centralization is closer to 1.

In centralization, the centrality of a central vertex is compared with that of an edge vertex. If a network is concentrated, the centrality of the central vertex must be high, and the centrality of the edge vertex must be low. By contrast, if a network is dispersed, there is no obvious difference in centrality between the central and edge vertices. Our result shows that the out-degree centrality is 2.885%, whereas the in-degree centrality is 16.492%, meaning that patients seeking online consultations show a better trend in terms of reasonable distribution because of more dispersed distribution than physicians who provide telephone consultation services. As these physician consultants construct a more centralized network, there is a uniform geographic distribution of patients who use OHCs, whereas the physician relies more on geographic advantage (eg, a higher medical resource capability).

**Table 1:** Statistical results of the social network analysis.

Province	Absolute out-degree centrality	Absolute in-degree centrality	Relative out-degree centrality	Relative in-degree centrality
Beijing	1292	22261	0.954	16.444
Shanghai	713	10343	0.527	7.64
Jiangsu	3652	1997	2.698	1.475
Guangdong	1647	1165	1.217	0.861
Tianjin	1350	922	0.997	0.681
Shandong	3426	905	2.531	0.669
Shaanxi	794	764	0.587	0.564
Henan	2087	558	1.542	0.412
Hubei	932	537	0.688	0.397
Hunan	963	476	0.711	0.352
Sichuan	772	294	0.57	0.217
Zhejiang	3783	293	2.794	0.216
Anhui	2099	290	1.55	0.214
Fujian	847	222	0.626	0.164
Liaoning	1695	199	1.252	0.147
Chongqing	357	177	0.264	0.131
Hebei	5010	125	3.701	0.092
Heilongjiang	1390	118	1.027	0.087
Shanxi	2506	67	1.851	0.049
Jiangxi	1032	64	0.762	0.047
Jilin	892	33	0.659	0.024
Yunnan	421	28	0.311	0.021
Guangxi	341	24	0.252	0.018
Gansu	684	22	0.505	0.016
Hainan	167	19	0.123	0.014
Inner Mongolia	1809	10	1.336	0.007
Guizhou	381	7	0.281	0.005
Xinjiang	434	4	0.321	0.003
Ningxia	259	1	0.191	0.001
Qinghai	163	0	0.12	0
Tibet	27	0	0.02	0

### Construction of the Regression Model

This study verifies the related hypothesis that OHCs will be influenced by offline medical resource distribution. The analysis in this section uses a regression model to fit the empirical data.

The dependent variable in the model represents the frequency of social exchange that happens via *TeleConsultation* in the OHC between any 2 provinces; for example, among the 92,492 telephone consultation records between physicians and patients crawled from the OHC, the provincial administrative region *i* where physicians are located can be matched using the information related to the hospital at which the physician works, and the region *j* where patients are located can be extracted from the detailed patient information. Subsequently, the statistics are measured by the teleconsultation frequency from region *j* to region *i*.

On the basis of our hypothesis, the independent variables are measured by the spatial accessibility of objective offline medical resources, the difference in their capital availability, and the interaction of accessibility and availability. Spatial accessibility refers to both the intensity and the possibility of interaction with medical resources [13], in terms of the distance between region *i* (the location of both the physician and the physician-patient social exchange) and region *j* (the patient location). Hypothesis 1 states that spatial accessibility has an inverted U–shaped effect on online physician-patient social exchange. Therefore, the squared variable (*distance^2^*) is introduced to fit the model. Capital availability also refers to the service capability of resources and is commonly measured by average resources per capita. In traditional public health research, capital availability is generally estimated by the number of beds in hospitals and health centers per 1000 population [13,29]. Therefore, this study uses *PerBedRate* to estimate the difference in the availability of offline medical resources, specifically the ratio of the number of beds in hospitals and health centers per 1000 population in provincial region *i* to the number of beds in hospitals and health centers per 1000 population in provincial region *j*. The interaction of spatial accessibility and capital availability *distance_PerBedRate* is measured by the product of *distance* and *PerBedRate*.

To narrow the focus to the functions of offline medical resources, the effect of physicians’ web-based efforts on the dependent variable needs to be eliminated. In this study, an internal index of the OHC, the contribution value, is used to measure the web-based performance of physicians. The contribution value is automatically calculated by the software system of our target platform using an empirical algorithm that is not publicly available. According to the brief introduction of the contribution value on our target platform, it is calculated based on the following 3 aspects: modifying personal information on the home page, publishing health care papers, and providing free consultations. The numerical value assigned to their contribution is displayed prominently on each physician’s home page and measures the physician’s web-based contribution, which patients can view directly. Patients understand the level of physicians’ web-based efforts by comparing the numerical values assigned to their contributions; however, they only know the meaning that the numerical value represents and do not clearly know the calculation method (such as the weight of each dimension), that is, the contribution value is the *score* obtained by physicians through their personal choices and efforts [36,37]. Hence, we estimate the control variable *contribution* by including a cumulative contribution value of all the physicians in province *i*. A description of all the variables in our model is listed in Table 2.

On the basis of these variables, we specify the regression model with the squared variable in formula (4):

log(*TeleConsultation*)=*β*_0_+*β*_1_*distance*+*β*_2_*distance*^2^+ *β*_3_*PerBedRate*+*β*_4_*distance*_*PerBedRate*+ *β*_5_log(*contribution*)+*ε* (4)

In this model, *ε* is a random error term. In addition, to eliminate collinearity, we take the logarithm of the dependent variable (*TeleConsultation*) and control variable (*contribution*). Moreover, we normalize the independent variable (*PerBedRate*). Table 3 shows the descriptive statistics of the variables in the model.

**Table 2:** Description of the variables in the regression model.

Type (variable)		Description
Dependent variable (TeleConsultation)		The frequency of web-based telephone consultations between 2 provincial administrative regions
**Independent variable: spatial accessibility**		
	Distance	The spatial distance between 2 provinces
	Distance^2^	The square of the spatial distance between 2 provinces
	Independent variable: capital availability (PerBedRate)	The number of beds in hospitals and health care centers per 1000 population
	Interaction variable (Distance_PerBedRate)	The interaction of accessibility and availability
	Control variable (contribution)	The total contribution value of a physician in a certain provincial administrative region

**Table 3:** Descriptive statistics of the variables.

Variables	Sample size	Values, mean (SD; range)
Log(TeleConsultation)	635	2.43 (1.88; 0 to 9.70)
Distance	635	972.05 (65.32; 0 to 658.63)
Distance^2^	635	1378219 (1662165; 0 to 9237601)
PerBedRate	635	0.00 (0.211; –0.78 to 0. 77)
Distance_PerBedRate	635	200.08 (10.36; –507.21 to 507.21)
Log(contribution)	635	0.31 (4.40; 0 to 15.06)

## Results

To provide clearer insights into the explanatory power of each variable with respect to the dependent variable, stepwise regression was used to fit the model. Model 1 contains only the control variable, whereas models 2 and 3 continually add the main effects and interaction to the model.

### Results of the Regression Models

The results of the regression models are shown in Table 4.

For models 1, 2, and 3, for a sample size of 635 each, the *R*^2^ values were 0.0632, 0.1405, and 0.1928, respectively, whereas the *F* change values were 298.732, 636.662, and 489.923, respectively.

Model 1 suggests that a physician’s online contribution significantly affects the frequency of physician-patient telephone consultations (β_contribution_=.210; *P*<.001). However, this model has weak explanatory power because it measures only 6.32% (*R*^2^=0.0632, *F*_9,627_=298.732) of the interrelationship of physician-patient telephone consultations in the OHC. Excluding the control variable, the addition of the main effect into the model (model 2) leads to a significant increase in explanatory power (*R*^2^=0.0773, *F*_8,628_=436.662). Spatial accessibility (distance) has an inverted U–shaped effect on physician-patient telephone consultations in OHCs (β_distance_=.199; *P*<.001 and β_distance_^2^=–.00449; *P*=.008). This result supports hypothesis 1. The frequency of web-based physician-patient interactions (via telephone consultations) is influenced by the spatial accessibility of offline medical resources, which shows an inverted U–shaped effect. The capital availability result shows that the difference in the number of beds in hospitals and health care centers between 2 regions positively influences physicians’ social exchange (β_bed_=.935; *P*=.07). Therefore, hypothesis 2 is verified. With the inclusion of the interaction, the explanatory power improves considerably (*R*^2^=0.0523, *F*_9,628_=489.923). The interaction between spatial accessibility and capital availability is confirmed, and the interaction coefficient is positive (*P*<.001). Thus, model 3 validates hypothesis 3: the spatial accessibility and availability of capital play complementary roles in influencing web-based physician-patient social exchange. In summary, all hypotheses are verified under a certain level of explanatory power.

**Table 4:** The results of the regression models.

	Model 1 (control variable)		Model 2 (adding the main effect)		Model 3 (adding the interaction)	
	β (SE)^a^	*P* value	β (SE)	*P* value	β (SE)	*P* value
Log(contribution)	.210 (5.29)	<.001	.182 (5.24)	<.001	.180 (5.20)	<.001
Distance	N/A^b^	N/A	.199 (5.48)	<.001	.202 (5.68)	<.001
Distance^2^	N/A	N/A	–.00449 (–3.25)	.008	–.0046 (–3.50)	<.001
PerBedRate	N/A	N/A	.935 (2.48)	.07	1.313 (3.46)	<.001
Distance_PerBedRate	N/A	N/A	N/A	N/A	.00342 (1.17)	.06
_Cons^b^	N/A	N/A	3.599 (18.02)	<.001	3.640 (18.26)	<.001

^a^Values in parentheses are the robust SEs.

^b^Cons: constant term.

### Robustness Checks

To increase the reliability of the test results of the initial model, our study used 2 types of robustness tests.

First, prior research shows a significant difference in the level of spontaneous consumption [38] and the efficiency of health input [29] between the northern and southern regions of China. Therefore, this study fits the model by separating the sample data by geographic location into the northern and southern provincial administrative regions to eliminate the influence of the difference between the northern and southern regions. The test results are shown in Table 5.

For check 1 (northern region), for a sample size of 330, the *R*^2^ value was 0.2011, whereas for check 2 (southern region), for a sample size of 306, the *R*^2^ value was 0.6723.

In general, the number of beds in hospitals and health care centers in the northern region has a nonsignificant (β_bed_=1.048; *P*=.83) influence on online physician-patient social exchange. All of the proposed hypotheses still hold, regardless of area. This result indicates that the availability of offline medical resources has no significant influence on decisions with regard to online physician-patient interactions. This finding may be caused by the generally low level of capital availability in north China because patients are already less sensitive to capital availability when seeing a physician. However, the validity of this speculation needs to be supported by additional empirical data. Moreover, the explanatory power of the model is significantly different between the northern and southern regions, and the goodness of fit of the model for south China is better than that for north China (*R*^2^_south_=0.6723>*R*^2^_north_=0.2011). It seems that most of the frequency of online physician-patient telephone consultations in the southern region can be explained by the distribution of offline medical resources, whereas in the northern region, there are other influential factors.

We used replacement variables to conduct a second robustness check. The number of general hospitals in public health research was used to measure spatial accessibility [13]. Therefore, this robustness check was conducted by replacing the difference in the number of beds in hospitals and health care centers per 1000 population with the difference in the number of hospitals (*HospitalRate*). Table 6 shows that most of the results are consistent with the initial empirical analysis results after replacing the variable. However, the capital availability and spatial accessibility variables are not complementary under the replacement variable. It is possible that when measuring the availability of capital, the variable *PerBedRate* used in the original model is more representative than the other indices, resulting in measurement bias. Overall, the analysis results of this study pass the robustness tests and are credible (for a sample size of 635, the *R*^2^ value was 0.1533).

**Table 5:** The robustness checks based on the classification of the samples.

	Check 1 (northern region)			Check 2 (southern region)	
	β (SE)^a^	*P* value	β (SE)		*P* value
Log(contribution)	.109 (2.70)	.004	1.050 (21.06)		<.001
Distance	.187 (3.75)	<.001	.213 (5.27)		<.001
Distance^2^	–.00389 (–2.18)	.07	–.00597 (–3.67)		<.001
PerBedRate	1.048 (1.59)	.83	0.886 (2.50)		.05
Distance_PerBedRate	.00976 (3.23)	.003	.00948 (3.13)		.006
_Cons^b^	3.736 (12.85)	<.001	–11.55 (–15.24)		<.001

^a^Values in parentheses are the robust SEs.

^b^Cons: constant term.

**Table 6:** The robustness check based on a replacement variable.

	Check 3 (replacement variable)	
	β (SE)^a^	*P* value
Log(contribution)	.106 (3.66)	<.001
Distance	.192 (5.26)	<.001
Distance^2^	–.00408 (–2.95)	.001
HospitalRate	1.027 (2.67)	.009
Distance_HospitalRate	.00870 (0.25)	.47
_Cons^b^	2.615 (5.09)	<.001

^a^Values in parentheses are the robust SEs.

^b^Cons: constant term.

## Discussion

### Principal Findings

This study explores the impact of the spatial accessibility and availability of offline medical resources on the social exchange between physicians and patients in OHCs based on online physician-patient telephone consultation data and offline health care capital distribution data. The empirical test results support the notion that physician-patient telephone consultations will be positively influenced by physicians’ online contributions and capital availability, and, interestingly, spatial accessibility has an inverted U–shaped effect.

This study offers theoretical support for the impact of offline channels on online channels. The results indicate that online physician-patient social exchange in OHCs will be influenced by the spatial distribution of offline medical resources. When researching online telephone consultations, the geographic distance from, and service capability of, offline resources should be considered. Internet medical services are not an entirely new channel that is independent of, and separate from, traditional medical health care. To a certain degree, OHCs still temporarily serve as an auxiliary function of offline medical health care, and their use remains constrained by offline health care capital.

In addition, this study provides a relevant theoretical basis for research on the equity of medical resources related to online channel support. Urban-rural health care inequity stems from unequal access to medical resources [39]. The existence of OHCs can reduce this inequity; for example, Goh et al [27] point out that, compared with rural users, urban users of OHCs are more likely to provide social support, which is a helpful mechanism for emotional support and medical knowledge to flow from urban areas to rural areas. Although offline medical services constrain online consultation, they still provide a channel for medical resources to be distributed from places with high availability to those with low availability. This kind of channel effect shows a more significant effect on online consultation as spatial accessibility and capital availability grow. Therefore, OHCs offer a new research direction and theoretical basis for studies related to eliminating the geographic imbalance of medical resources caused by differences in economic development and population distribution.

### Limitations and Further Directions

Although this study has made significant contributions through this empirical research, it includes some limitations in terms of the research scope and methods. First, the data in this study come from China. Whether the results can be extended to other regions may depend on the differentiated medical systems and OHC development in these regions. Future cross-regional studies will help to explore the generalization and extensibility of the results of this study. Second, because of the privacy protection settings of our target OHC, although we have inferred the geographic location of the patients through IP calculation, we still cannot accurately locate the online consultation interaction of patients. Therefore, detailed data obtained from different data sources will help to make the research conclusions more accurate.

### Implications for Practice

The relevant findings of this study have important implications for the managements of OHCs and the government policy–making process. First, the results show that online consultations are not independent of offline treatment and that these online consultations are influenced by offline health care capital distribution. Hence, the significant impact of the allocation of offline medical resources on online physician-patient social exchange should be confronted by OHC managements. Geolocation-based referrals can be used to recommend physicians who are farther away from the locations of patients because through this measure, more appropriate physicians can be distributed to patients who are searching for physicians online. Moreover, other research findings for government policy makers demonstrate that the emergence of internet health care (eg, OHCs) can compensate for geographic distance–induced health care deficiencies. In fact, prior research has demonstrated that OHCs reduce the rural-urban disparities in the availability of institutional forms of health care capital (eg, emotional support) [27]. This study verifies that the use of OHCs can compensate for the insufficient spatial accessibility of offline medical resources and direct resources from areas with high capital availability to those with low availability. Indeed, this form of online medical care has reduced geographic limitations and promoted the flow of medical resources across regions. Finally, the complementarity of spatial accessibility and capital availability is verified under the premise that the spatial accessibility of medical resources has an inverted U–shaped impact on online physician-patient social exchange. When the target area’s capital availability is increased, the inflection point of spatial availability will shift to the right, thus expanding the available range for patients.

### Conclusions

This study explores the relationship between online and offline channels by investigating online physician-patient interactions and offline medical resources. Offline capital distribution and online physician-patient interactions are used to explore the impact of medical resources on physician-patient social exchange in OHCs. This study demonstrates that online physician-patient interactions are influenced by spatial accessibility and capital availability of offline medical resources. This study creatively analyzes the influence of the offline channel on the online channel and verifies the possibility that capital can be transferred from high-availability regions to low-availability regions by using OHCs. Moreover, it complements the literature on the impact of offline medical resources on online physician-patient social exchange and offers theoretical support for OHC research and the distribution of medical resources, as well as online and offline channels. The related results are also used to make sensible recommendations for practical applications to be used by OHC managements, governments, and other stakeholder organizations.

## Abbreviations

OHC: online health community

## Data Availability

The data analyzed in this study are publicly available. The offline data were obtained from the China Health Statistical Yearbook 2014; the online data can be obtained from Haodf (using a web crawler will reduce the time spent on data acquisition).
